# THE IMPACT OF COVID-19 CONCERNS ON OLDER ADULTS’ DEPRESSION: THE ROLES OF RACE/ETHNIC STATUS AND EDUCATION

**DOI:** 10.1093/geroni/igad104.3416

**Published:** 2023-12-21

**Authors:** Shan Qu, Jeffrey Burr

**Affiliations:** University of Massachusetts Boston, Boston, Massachusetts, United States; University of Massachusetts Boston, Boston, Massachusetts, United States

## Abstract

Framed within the Conservation of Resources Theory and the Cumulative (Dis)advantage Theory, this study examined the association between COVID-19 concerns (worries) and risk of depression among older adults. The study also investigated whether race/ethnic status and education moderated this relationship. We used a nationally representative sample of Americans over age 50 from the 2020 Health and Retirement Study. Depression was defined as having greater than or equal to three depressive symptoms, as derived from the 8-item Center for Epidemiologic Studies-Depression scale. COVID-19 concerns were measured with respect to respondents’ own health, the health of family members, respondents’ financial situation, the ability to get help from family, friends, or others during the pandemic, and concerns about the future; the study also included a summative index of these concerns. The associations between COVID-19 concerns and older adults’ depression were estimated with unadjusted and adjusted logistic regression models. Compared to respondents with lower scores on the COVID-19 concern measures (the index and the five domains), those with higher scores were more likely to be depressed. The moderation models showed the relationship between respondents’ concerns about what will happen in the future and depression was not as strong among non-Hispanic Black older adults compared to non-Hispanic White older adults. There was no statistically significant moderation effect for education. The results imply that healthcare providers should focus on vulnerable older adults to reduce disparities in access to resources during public health emergencies like the COVID-19 pandemic.

